# The nine hallmarks of mental health and wellbeing as a watershed framework for resilient living and healthspan optimization

**DOI:** 10.3389/fpsyg.2026.1749193

**Published:** 2026-04-10

**Authors:** Isabel Garcia-Garcia, Justin Carrard, Jean-Bernard Daeppen, Patrizia D’Amelio, Simone Gibertoni, Adrian Heini, Julijana Ivanisevic, Ernst T. Rietschel, Goranka Tanackovic, Olga Donica

**Affiliations:** 1 Clinique La Prairie, Clarens-Montreux, Switzerland; 2 Sport and Youth Health Unit, Children and Adolescent Surgery, Women’s, Maternal and Child Health Department, Lausanne University Hospital, Lausanne, Switzerland; 3 Sport and Exercise Medicine Center, Department of Musculoskeletal Medicine, Lausanne University Hospital, Lausanne, Switzerland; 4 Institute of Sport Science, University of Lausanne, Lausanne, Switzerland; 5 Division of Sport and Exercise Medicine, Department of Sport, Exercise and Health, University of Basel, Basel, Switzerland; 6 Addictions Medicine, Lausanne University Hospital, University of Lausanne, Lausanne, Switzerland; 7 Service of Geriatric Medicine and Geriatric Rehabilitation, Lausanne University Hospital, Lausanne, Switzerland; 8 Metabolomics Unit, Faculty of Biology and Medicine, University of Lausanne, Quartier UNIL-CHUV, Lausanne, Switzerland; 9 Department of Epidemiology and Health Systems (DESS), University Center for Primary Care and Public Health, Lausanne, Switzerland

**Keywords:** cardiovascular, chronic stress, fatigue, gut-brain axis, immune, mental health, social network

## Abstract

Mental health and wellbeing are core components of a healthy life. However, emotional stress and mental health problems are on the rise through societies and cause a significant burden of suffering. In the following, we present our watershed framework for optimizing wellbeing and mental health through lifestyle interventions. Emerging evidence also shows that mental health is an important determinant of quality of life. In addition, it may also be a driver of longevity, influencing health outcomes and, ultimately, the overall healthspan. This framework is based on the recognition that mental health is comprised of biological, psychological, and social factors, and that the factors have interdependent relationships amongst them, so that positive changes in one of them can have cascading effects on the others, with the potential to improve the psychological capital of an individual. Our framework consists of nine elements that we consider hallmarks of mental health and wellbeing, structured in three interdependent layers. The first layer is formed by foundational factors, which are essential for wellbeing and mental health, including circadian rhythms and sleep recovery, along with social connections. The second layer, named wellbeing catalysts, encompasses elements of wellbeing that can have powerful positive effects on mental health, including stress resilience, vitality, cardiovascular health, immune health, and the gut-brain axis. The third layer, named as thriving factors, consists of cognitive performance and positive emotions, which are the elements most often associated with a thriving mind. In our framework, we highlight the interactions between the factors and provide a roadmap to structure comprehensive lifestyle interventions aimed at optimizing mental health and wellbeing. Strengthening mental health and wellbeing addresses key biological and behavioral determinants of healthy aging, which are increasingly recognized as critical modulators of longevity trajectories.

## Introduction

Mental health and wellbeing are intimately associated with a healthy life, although their causality links remain unclear (Song et al., 2023; Zaninotto and Steptoe, 2019). For instance, a prospective study that included over 9,500 participants from the English Longitudinal Study on Ageing (ELSA) and with an average follow-up of 6 years showed that people with high levels of subjective wellbeing at baseline had a longer lifespan and a longer life without disability relative to people with lower subjective wellbeing (Zaninotto and Steptoe, 2019). Another study has estimated that having strong social connections, a fundamental component of wellbeing, can increase the likelihood of survival by 50% over a 7 year follow-up, independent of health status and gender (Holt-Lunstad et al., 2010). This effect is particularly significant among older adults, who are especially vulnerable to the risks of loneliness and social isolation. In a large cohort of veterans discharged to skilled nursing facilities after heart failure hospitalization, those with high social connectedness were 21% more likely to experience a successful discharge compared to those with lower social connectedness. The impact was even greater among cognitively impaired patients (Chen et al., 2025). Together, these findings highlight the strong connections between wellbeing and longevity.

One of the big threats to mental health and wellbeing is psychosocial stress. There is a worsening of emotional stress that affects the worldwide population and spans over the last decades. In 2007, around 26% of the population reported experiencing emotional stress, while the same figure reached approximately 35% in 2017, and it has stayed approximately the same ever since (Piao et al., 2024). This rise in emotional stress appears to be independent of the type of residential area (rural/farm, small town/village, large city, and suburban areas) and employment status (full-time, part-time, self-employment, or unemployment) assessed (Piao et al., 2024). Similar to what is observed in emotional stress, mental health problems are also growing in numbers. Between 35 and 50% of people in need of treatment for mental health do not receive any kind of intervention or therapy. This alarming figure was shown in a report by the executive board of the World Health Organization (WHO) from 2011. The report was a call for action about the urgent need for a coordinated response by healthcare and social services. The rise of mental health problems, and especially affective disorders, was accentuated by the COVID-19 pandemic. It also seems to have a particularly strong impact among women and younger age groups (Santomauro et al., 2021).

Just as wellbeing is associated with a longer healthspan, emotional stress is intricately related to worse health outcomes. For instance, one of the most important contributing factors to emotional stress is physical pain. Individuals who experience pain show between 14 and 20% increases in emotional stress (Piao et al., 2024). While people with health problems also tend to have worse psychological wellbeing, as a group they show increases in emotional stress that range between 1.5 and 2.6%, numbers that are substantially lower than the ones observed for physical pain (Piao et al., 2024). There is also some evidence that daily stress may precede the occurrence of certain health symptoms. This includes sore throat, headache and back pain, as shown by a highly-cited study that assessed data over a 6 month period (DeLongis et al., 1988).

Mental health and wellbeing are fundamental in the pursuit of a healthy life. At the same time there is a persistent increase in stress and mental health problems in our society. These two facts set the context for the need to develop strategies that tackle the prevention of mental health problems and the optimization of wellbeing. It is in this context that we propose the Watershed Framework of Mental Health and Wellbeing. This framework aims at providing a roadmap for lifestyle-based interventions to improve mental health and wellbeing. Our framework hypothesizes that specific elements of wellbeing and mental health can have positive cascading influences over the rest, hence the name of watershed. In the following, we develop our framework, and we highlight lifestyle interventions that can be deployed to provide an integrated effort aimed at optimizing physical and psychological health and wellbeing.

As a note on the scope of this article, here we are presenting an original theoretical framework rather than a systematic review. For this reason, sources were not identified through a comprehensive, protocol-driven search characteristic of systematic reviews. We have instead selected the literature to justify the inclusion of nine specific wellbeing domains and to explain their interrelations within the proposed framework.

### Mental health extends beyond the absence of mental disorders and it is biopsychosocial in nature

The Watershed Framework of Wellbeing and Mental Health has been nurtured by two main sources of inspiration:

First, we adopt the view of the World Health Organization, which considers that mental health has an intrinsic value, it is core to wellbeing, and it is an essential component of health. The definition that the World Health Organization provides about mental health is the following:

*“Mental health is a state of mental well-being that enables people to cope with the stresses of life, realize their abilities, learn well and work well, and contribute to their community. It is an integral component of health and well-being that underpins our individual and collective abilities to make decisions, build relationships and shape the world we live in. Mental health is a basic human right. And it is crucial to personal, community and socio-economic development”* (text extracted from www.who.int and consulted in March 2025).

According to the definition by the World Health Organization, mental health extends beyond the mere absence of mental disorders and expands along a continuum. Individuals differ in the degree of difficulty and distress that they experience. For this reason, adherence to a *reactive* model of mental health and wellbeing, which intervenes only when disorders are present, has inherent limits in its impact. A large segment of the population may experience suboptimal mental health and live with a significant daily stress and suffering while not meeting strict clinical criteria for a specific mental disorder. Strategies to promote and protect mental health and wellbeing should be addressed to everybody, not just limited to patients with clinical diagnoses (World Health Organization, 2022).

Second, we adopt the view that Wellbeing and Mental Health are profoundly influenced by psychological, physiological, and social factors that collectively shape an individual’s overall function. This line of thought was developed by George Liebman Engel in his Biopsychosocial model of illness and healing published in 1977 (Engel, 1977). Engel argued that health outcomes result from the dynamic interplay of these three domains, moving beyond the traditional biomedical approach. According to this view, biochemical alterations associated with medical diseases only indicate disease potential, far from reflecting the actual state of the disease and its impact for the patient. To understand how the disease is experienced and how it affects the individual, psychological, social, and cultural factors need to be considered. Taking the example of diabetes, the laboratory confirmation of altered blood glucose or HbA1c levels provides a partial account of the disease. Psychosocial factors need to be taken into consideration to fully understand the expression of the illness and the experience of the individual. Likewise, Engel defended that mental health and mental problems need to be understood by taking into account the three perspectives: biological, psychological, and social (Engel, 1977). The Watershed Framework of Wellbeing and Mental Health is profoundly influenced by this model, emphasizing the importance of designing assessments and interventions that address biological, psychological, and social factors.

## The nine hallmarks of mental health and wellbeing in a watershed framework

**Figure 1:**
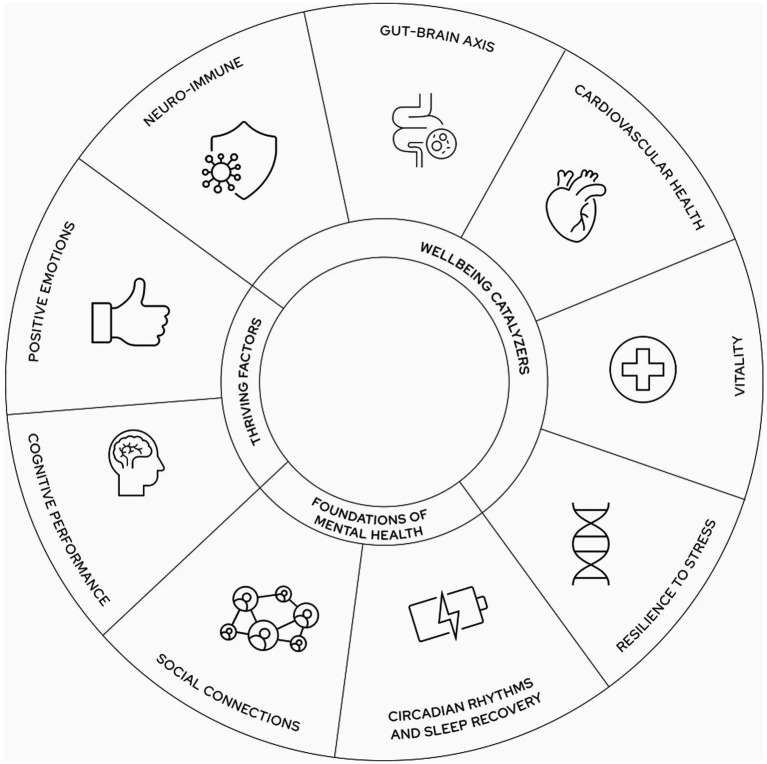
The nine elements of the watershed framework of wellbeing and mental health.

The Watershed Framework of Mental Health and Wellbeing is built around the nine elements that we consider as hallmarks of mental health and wellbeing: social connections, circadian rhythms and sleep recovery, resilience to stress, vitality, cardiovascular health, neuro-immune health, gut-brain axis, positive emotionality, and cognitive performance (Figure 1).

In our model, mental health and wellbeing are the result of a highly interconnected system, where biological, psychological, and social dimensions influence one another. These influences are conceptualized in three interdependent layers, where a disruption in one element often cascades into others. With this multidimensional perspective, we want to emphasize that meaningful long-lasting improvements in mental health require attention to each layer, that is, by addressing both the roots and the expressions of wellbeing and mental health.

The first layer, *Foundational Elements*, comprises those aspects of wellbeing that have the greatest influence on multiple other dimensions of mental health and overall functioning. We have identified circadian rhythms & sleep recovery along with social connections as key foundational elements because their disruptions tend to propagate across different psychological and physiological domains. As an example of this, lack of sleep may potentially compromise resilience to stress, vitality, cardiovascular health, immune health, and cognitive performance.

The second layer, known as *Wellbeing* Catalysts, involves intermediate factors that can be considered outcomes of the foundational elements. We call them *catalysts* because we hypothesize that they can accentuate meaningful wellbeing changes on the other elements of wellbeing, rather than just constitute passive outcomes. Of note, although positioned in the second layer, they may also have an active influence on the Foundational Elements of our framework, and we will elaborate on this possibility later. In our framework, optimization of wellbeing catalysts is desirable not only as a goal, but also as a means to enhance the elements of the third layer: thriving factors. Resilience to stress, vitality, along with cardiovascular health, immune health, and the gut-brain axis are considered here as Wellbeing Catalysts. Being intermediate factors, Wellbeing Catalyst are partially dependent on foundational elements (e.g., both sleep and social connections may influence stress resilience). Conversely, they can also influence other domains of wellbeing and mental health (e.g., resilience to stress may impact attention, a component of cognitive performance).

**Figure 2:**
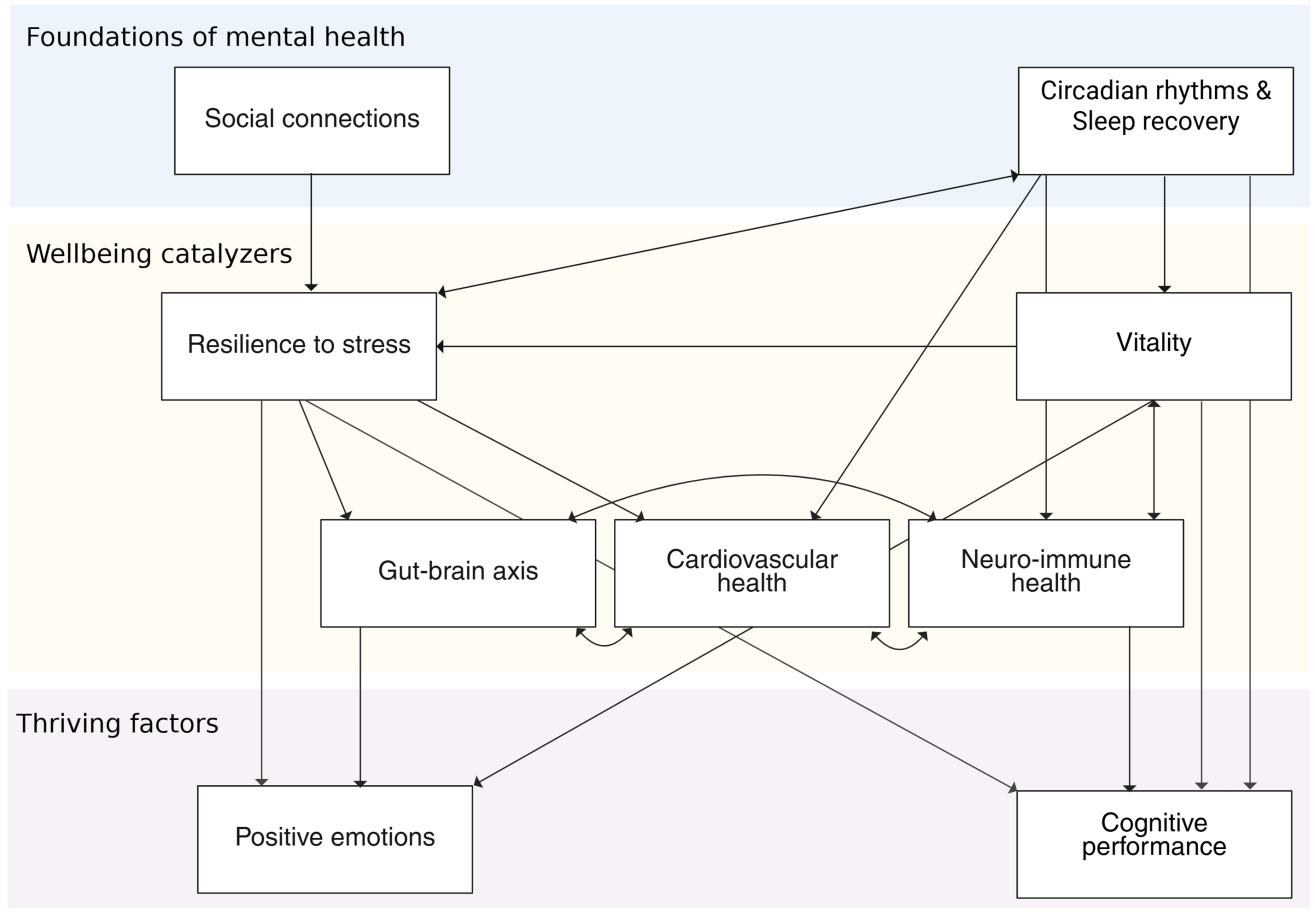
The watershed model of wellbeing and mental health, depicting the hierarchical relationships between elements. This hierarchical structure should be considered heuristic and hypothesis-generating, rather than empirically established. While most arrows are unidirectional, some dimensions are most likely influencing each other and are depicted with a bidirectional arrow. Figure created with BioRender.

The third layer, known as *Thriving Factors*, is influenced by the factors included in the Foundational Elements and the Wellbeing Catalysts. They can be regarded as outcomes of the upstream elements of the framework. This last layer represents positive and convergent outcomes of wellbeing and mental health, and its elements closely align with popular conceptions of what constitutes a flourishing mind. Put in other words, they reflect optimal functioning. These elements are highly relevant for mental health and general functioning. Here, we consider that positive emotionality and cognitive performance are thriving factors of wellbeing and mental health (Figure 2).

## Foundational elements of wellbeing: circadian rhythms and social connections

### Sleep and circadian rhythms

Circadian rhythms are endogenous biological oscillations with a period of about 24 h that allow organisms to anticipate and adapt to the daily light–dark cycle generated by the Earth’s rotation on its axis (Walker et al., 2020). Sleep-awake status, body temperature, the secretion of certain hormone levels, such as cortisol and melatonin, and the concentrations in plasma glucose are all examples of biological factors that follow circadian rhythms, and they show dynamic changes across the lifespan (Hood and Amir, 2017). Circadian rhythms are necessary to establish consistent wake and rest patterns, allowing the organisms to control metabolic needs and optimize energy production, as well as to have reliable rest periods to restore and repair the health of the cells and tissues. The suprachiasmatic nucleus, a small nucleus located in the hypothalamus, is responsible for synchronizing the different circadian rhythms (Kondratova and Kondratov, 2012). Circadian rhythms sustain mental health, and disruptions in circadian rhythms have been associated with psychiatric diseases, including affective disorders such as major depressive disorder, seasonal affective disorder, bipolar disorder, and anxiety disorders, along with schizophrenia (Walker et al., 2020). Conversely, early-morning exposure to bright light therapy seems to ameliorate symptoms in seasonal affective disorder, major depressive disorder, and bipolar disorder (Dollish et al., 2024). With regards to mood disorders, alterations in sleep-awake cycles are a core symptom of these disorders, and it has also been observed that acute changes in sleep–wake cycles can trigger mood episodes (Dollish et al., 2024; Walker et al., 2020).

Sleep is fundamental for our health and wellbeing. Despite this, sleeping problems are very common, with around 20–30% of adults reporting insomnia symptoms (Zheng et al., 2018). There are some life circumstances that substantially increase our risk of insomnia, such as being pregnant, having a baby, or suffering from depression. Another factor that increases the risk of sleeping problems is ageing. Sleep changes as we grow older, and some of these changes imply that people need a longer time to fall asleep, and their sleep becomes shorter in duration and more fragile. This last point means that they tend to wake up more often and that they experience more transitions to phases of non-REM light sleep. Older adults also spend less time in deep phases of non-REM sleep (known as slow-wave phases) (Mander et al., 2017). Sleep recovery plays a critical role in cognitive and emotional regulation, as restorative sleep is essential for brain function, and more specifically, for memory consolidation and stress resilience (Lo Martire et al., 2024; Zhang et al., 2022).

Having an occasional night of bad sleep is probably harmless. However, sustained sleep deprivation has negative health consequences. Sleep disturbances are a risk factor for Alzheimer’s disease and other dementias (Xu et al., 2020). A study using the Whitehall II cohort examined sleeping habits in people aged 50, 60, and 70 years old. Twenty-five years later, their incidence of general dementia was recorded. The study showed that participants who regularly slept 6 or fewer hours per night at baseline had a 30% increase in their risk of dementia compared with those who reported sleeping 7 h or longer regularly (Sabia et al., 2021). Another study followed a cohort of participants for 11 years and showed that participants who slept less than 6 h per night had a 29% increase in their risk of cerebrovascular disease (Vgontzas et al., 2013).

There are several standardized self-reported questionnaires that evaluate sleep. Examples of them are the Pittsburgh Sleep Quality Index (Buysse et al., 1989), the Sleep Quality Scale (Yi et al., 2006) or, when insomnia is present, the Insomnia Severity Index (Morin, 1993). Objective evaluations of sleep range from the traditional and gold-standard polysomnography to wearable sleep monitoring devices, which differ in accuracy depending on the technology (Chen et al., 2023; Lu et al., 2024).

In people with chronic insomnia, recommended behavioral and psychological treatments comprise cognitive-behavioral therapy for insomnia, stimulus control, which is an intervention aimed at restoring the association of bed with sleep, sleep restriction therapy, and relaxation training and mindfulness (Edinger et al., 2021).

In non-clinical populations, sleep hygiene routines usually consist of recommendations such as avoiding large meals for dinner, going to bed at a constant time, screen reduction or elimination, reducing bedroom noise, and reducing caffeine intake. While there are some inconsistencies in how effective each of these routines is in optimizing sleep, maintaining a consistent sleep schedule, and avoiding the use of screens are amongst the most scientifically supported guidelines for better sleep (Irish et al., 2015). Along with these recommendations, reducing or eliminating alcohol consumption is also important to improve sleep quality, since even low doses of alcohol can reduce REM sleep, while higher doses also shorten sleep onset latency and latency to deep sleep (Gardiner et al., 2025).

Vagus nerve stimulation is emerging as a potential intervention to improve sleep quality and ameliorate symptoms of insomnia (de Oliveira et al., 2025). Among the non-invasive modalities of vagal stimulation, transcutaneous auricular vagus nerve stimulation is gaining traction because of its safety, non-invasiveness, and portability (Redgrave et al., 2018). Initial clinical studies are showing that transcutaneous vagus nerve stimulation increases sleep quality and improves symptoms of insomnia (de Oliveira et al., 2025; Yeom et al., 2025), supporting the potential of vagus nerve stimulation in promoting rest and optimizing sleep.

### Social connections

Social connections encompass the feelings of belonging to a group or a community, as well as the sense of emotional closeness. They provide crucial emotional and practical support, reinforcing psychological resilience and mitigating the adverse effects of stress and loneliness. The counterpart of social connections is loneliness, a feeling that can cause such profound psychological pain that underscores the fact that social connections are one of the most fundamental pillars of wellbeing and mental health (Cacioppo and Cacioppo, 2018; Seligman, 2011).

Examples of standardized scales that can be used to measure social connections are the Social Connectedness Scale (Lee and Robbins, 1995), the 3-items Loneliness Scale (Hughes et al., 2004), and the Social Network Index (Cohen et al., 1997).

A comprehensive meta-analysis of 148 studies found that individuals with stronger social relationships had a 50% increased likelihood of survival compared to those with weaker ties. This effect remained significant across various age groups, health status, and causes of death (Holt-Lunstad et al., 2010). A more recent study analyzing the UK Biobank database and including over 450,000 participants found that both functional (e.g., frequency of confiding in someone) and structural (e.g., living alone) aspects of social connection were independently associated with all-cause and cardiovascular mortality (Foster et al., 2023). Another study that included data across 19 countries examined changes in social connections during older adults’ last years and found that stronger social ties were associated with better end-of-life outcomes, including fewer symptoms and reduced healthcare utilization. This result emphasizes the importance of social connections on the healthspan (Pivodic et al., 2024).

Group-based programs, such as community centers or clubs, seem to effectively reduce social isolation and improve mental health among older adults (Suragarn et al., 2021). Engaging in volunteering work also fosters a sense of purpose and community belonging, this way strengthening social ties (Smith et al., 2023). By fostering a positive connection with oneself, society, and nature, mindfulness-based interventions may also enhance prosocial feelings, such as compassion, and may help reduce prejudices, anger, and retaliation (Zhang et al., 2021). Finally, self-care strategies can improve self-regulation and confidence, facilitating positive social relationships (Jordan, 2023).

## Wellbeing catalyzers: resilience to stress, vitality, cardiovascular health, immune health, and the gut-brain axis

### Resilience to stress

Resilience to stress is the component of mental health that enables individuals to adapt and recover from adversity, stress, and negative life events while maintaining emotional stability. Deficits in this dimension can manifest as psychological stress, and its symptoms might include feelings of sadness, anxiety, fear, anger, guilt, irritability, or hopelessness, as well as physical symptoms such as sleep alterations, appetite disturbances, or lack of vitality, among many others. Resilience to stress is multifactorial, with multiple biological, psychological, social, and ecological factors influencing this dimension (Ungar and Theron, 2020) (Table 1). More specifically, regarding psychological factors, a study evaluated what psychological constructs were associated with good mental health in the face of different stressors caused or worsened by the COVID-19 lockdown in 2020. The results showed that positive appraisal, or the tendency to reframe stressors in a more positive manner, was linked with resilience. Positive appraisal also acted as an intermediate factor in the relationship between perceived social support and resilience (Veer et al., 2021).

**Table 1:** Multiple factors influence resilience to stress.

Systems influencing resilience	Examples [as reviewed in Ungar and Theron (2020)]
Biological	Genetic factors (e.g., serotoninergic polymorphisms), endocrine factors (e.g., responsivity of the HPA axis), epigenetic factors
Psychological	Secure attachment, self-esteem, positive appraisal
Social	Social network, social cohesion, community support, employment, mother’s mental health
Ecological	Quality of the school environment, housing, quality of the neighbourhood, preservation of natural spaces

Psychological stress has been associated with different causes of mortality in a dose-dependent manner (Russ et al., 2012). More specifically, chronic mental stress is associated with a harmful cascade of vascular alterations, which include increases in vascular inflammation, the development or worsening of the atherosclerotic plaques, and endothelial dysfunctions (Sara et al., 2022). High levels of stress are also associated with poorer sleep quality (Alotaibi et al., 2020).

By contrast, some studies suggest that mental health resilience is protective against mortality. In this regard, Zhang et al. (2024) analyzed data from the Health and Retirement Study database, which included over 10,000 participants aged 50 years or older. Participants in the study completed the Leave Behind Questionnaire, which provides an estimation of psychological resilience, and their incidence of all-cause mortality was recorded during a mean follow-up period of 12 years. The authors found that high resilience was protective against mortality: after controlling for potential confounders, people who obtained high scores in resilience had a 38% decrease in their risk of mortality compared to people scoring low in resilience (Zhang et al., 2024). These findings were corroborated in another cohort of older participants (average age 84 years old). Over a 3 year follow-up period, participants scoring high in a 5-item questionnaire about resilience had a 26% reduction in their risk of all-cause mortality relative to participants scoring low on resilience (Wang et al., 2024). Resilient individuals may be more likely to adopt healthy lifestyle behaviours, including being physically active, eating a balanced diet, or adhering to medical advice when needed. All these behaviours may be crucial to protect and optimize health, especially in older ages.

Resilience to stress can be measured or approximated through the following questionnaires: the Connor-Davidson Resilience Scale (Connor and Davidson, 2003), the Resilience Scale for Adults (Friborg et al., 2012), or the Brief Resilience Scale (Smith et al., 2008). With regards to objective measurements, heart rate variability has been postulated as a potential indicator of a person’s capacity to adapt to stressors in a flexible and adaptive manner, supporting the use of this indicator as a proxy of stress resilience (Perna et al., 2020).

Interventions to improve resilience to stress include mindfulness-based therapies, whose beneficial effects on wellbeing have been solidly established in both clinical and non-clinical populations (Petrova et al., 2021; van Agteren et al., 2021; D. Zhang et al., 2021). Mindfulness-based therapies are interventions focused on meditation, and their techniques typically include diaphragmatic breathing, mind–body scans, or the use of mindful imagery (van Agteren et al., 2021). Other psychological interventions such as cognitive-behavioral therapy, which target maladaptive thinking patterns and the improvement of coping skills and emotional regulation, are also clearly effective in improving wellbeing in populations with mental health disorders (van Agteren et al., 2021). Meditative practices that rely on paced breathing, or a voluntary slowing down of breath frequency, are also effective coping mechanisms to reduce arousal, anxiety, depression, anger, and confusion (Zaccaro et al., 2018).

### Vitality

Sustained energy levels and physical robustness characterize vitality. It is closely linked to both mental and physical health, influencing motivation and engagement in daily activities. Fatigue is the counterpart of vitality. Fatigue can be defined as the feeling of being extremely tired, usually as a result of hard physical or mental work. One out of five adults reports experiencing fatigue (Yoon et al., 2023), making it a highly prevalent health concern.

Vitality and fatigue are closely linked to general health. Research indicates that low vitality and persistent fatigue are associated with reduced quality of life, diminished daily functioning, and increased societal costs. For instance, a study using the National Health and Wellness Survey (NHWS) database, which includes over 24,000 participants living in five countries of the European Union, highlighted that individuals with low vitality experienced significant impairments in daily activities, mental health, and work productivity (Tardy et al., 2023). Another study has found that fatigue is associated with ischemic heart disease and mortality in non-smoking middle-aged men free of cardiovascular disease (Ekmann et al., 2013).

A standardized questionnaire to measure vitality is the Subjective Vitality Scale (Ryan and Frederick, 2019), while several other tools provide measurements of fatigue, including the Fatigue Assessment Scale (Michielsen et al., 2003), the Fatigue Severity Scale (Krupp et al., 1989), or the Bristol Rheumatoid Arthritis Fatigue Multidimensional Questionnaire (Dures et al., 2013).

A meta-analysis of 81 randomized-controlled trials has shown that physical exercise interventions can increase energy levels and decrease fatigue. Specifically, they showed that exercise decreases the feelings of fatigue by a small effect size, increases energy levels by a small-to-moderate effect size, and increases the feelings of vitality by a moderate effect size (Wender et al., 2022). The effects were dose-dependent, with vigorous and moderate-intensity exercises being more effective than light-intensity exercises. The authors also found that, in general terms, the longer the training session, the more impactful the results were (Wender et al., 2022).

In addition to physical activity, other interventions that may improve fatigue and optimize vitality include progressive relaxation, Tai-Chi, and cognitive behavioral therapy (Ho and Ng, 2020). Paced breathing has also been suggested to increase vigor and alertness (Zaccaro et al., 2018).

### Cardiovascular health

Cardiovascular health refers to the optimal functioning of the heart and the blood vessels, and represents a key determinant of overall health. Cardiovascular health and mental health are interrelated, with each influencing the other in a complex, bidirectional manner. Strikingly, mental health problems, such as psychosocial stress or anxiety, and cardiovascular disorders have several symptoms in common, including shortness of breath, palpitations, or chest tightness (Chaddha et al., 2016).

In 2008, the American Heart Association included depression as a major risk factor for coronary heart disease and highlighted the association between depression and cardiovascular morbidity and mortality (Lichtman et al., 2008). Adults with depression and without prior cardiovascular diseases have an 80% increase in the risk of suffering cardiovascular disorders and cardiovascular death (Chaddha et al., 2016). This association extends to other psychiatric disorders as well, such as eating disorders, and it seems that the risk of suffering cardiovascular diseases is particularly high during the first year after the diagnosis of a psychiatric disorder (Shen et al., 2023).

Early life stressors, such as maltreatment, or childhood socioeconomic adversity, are also associated with an increase in cardiovascular disease in adulthood (Steptoe and Kivimäki, 2013). Both exposure to acute traumatic stress and cumulative exposure to daily stressors can increase the risk of cardiovascular disease (Levine et al., 2021). In the case of stress, psychosocial stress, anger, and depressed mood can act as acute triggers of major cardiac events, with case cross-over studies estimating that acute stress is associated with a 2.5-fold increase in the relative risk of acute coronary syndrome, suggesting a significant impact of stress on cardiovascular health (Steptoe and Kivimäki, 2013). Work-related stress is also associated with a 40% increased risk of cardiovascular disease (Levine et al., 2021).

Some of the mechanisms by which affective symptoms can impact cardiovascular health are outlined in the following table (Chaddha et al., 2016).

**Table:** 

Mechanisms underlying the effect of affective symptoms on cardiovascular health
Increases in unfavourable lifestyle behaviors (e.g., smoking and alcohol intake, increased consumption of food rich in sugar and saturated fat, reduced medication compliance, physical inactivity)
Worsening of atherosclerosis
Hormonal dysregulations, which can increase resting heart rate and blood sugar levels
Increases in blood pressure

If mental health indicators such as stress or hostility traits have been associated with an increase in the incidence of cardiovascular disease, positive wellbeing factors have also been associated with a protection from cardiovascular pathologies. For example, a stronger sense of purpose in life has been linked to better cardiovascular health and a lower risk of cardiovascular disease (Levine et al., 2021). Likewise, higher scores in optimism are associated with a 35% reduction in cardiovascular events. These associations may be partly explained by the fact that individuals with greater wellbeing are more likely to engage in healthy behaviors, such as exercising more frequently and maintaining a healthier diet (Levine et al., 2021).

Cardiovascular health also has a strong influence on mental health. Cardiovascular health is fundamental for brain health and for cognitive performance (García-García et al., 2023). Along these lines, a very recent study has suggested that good cardiovascular health scores are associated with diminished blood levels of neurofilament light chain, a marker of neurodegeneration (Dhana et al., 2025). At the same time, cardiovascular diseases represent a major psychological burden for the patients, and a diagnosis of cardiovascular disease may lead to hypervigilance, constant worries about health, and feelings of being helpless. It can also lead to sleep problems and interfere with social activities (Borkowski and Borkowska, 2024). Major depressive disorder is frequently diagnosed following an acute myocardial infarction and in individuals with chronic heart failure, and this prevalence is estimated to be around 15–20% (Parlati et al., 2024).

Lifestyle interventions that have been shown to optimize and protect cardiovascular health, in either preclinical or clinical studies, are regular physical activity, nutrition patterns such as anti-inflammatory compounds, caloric restriction, and engaging in sleep hygiene patterns (Liberale et al., 2025).

Certain nutrients may also show protective cardiovascular effects, such as supplementation with NAD + precursors, and the so-called caloric restriction mimetics, including spermidine, resveratrol, and curcumin (Liberale et al., 2025). Medical interventions to protect cardiovascular health include managing blood pressure, cholesterol, and blood glucose levels through medications such as antihypertensives, statins, and antidiabetic drugs when needed (Liberale et al., 2025).

### Neuro-immune health

Immune health refers to the optimal functioning of the immune cells and immune responses, while neuro-immune health describes the strong links between immunity and brain functions, including mental health. Neuroinflammation, on the other hand, refers to the chronic activation of immune processes within the central nervous system (Walker et al., 2022). Neuroinflammation has emerged as a key factor linking chronic inflammation, ageing, and neurodegeneration. Under conditions of systemic inflammation, the chronic activation of microglial cells in the central nervous system can exacerbate the release of proinflammatory cytokines, oxidative stress, and neuronal injury, contributing to neurodegenerative pathology (Wilson et al., 2023). More specifically, in the field of mental health, psychoneuroimmunity is the examination of how psychological, neural, and immunologic processes are interrelated with each other and how their mutual synergies affect thoughts, emotions, and behaviors (Slavich, 2020). An example of how psychological, neural, and immune mechanisms work together has been illustrated by a recent publication examining responses to virtual infections; and they did so by showing participants virtual reality infectious avatars and comparing them with neutral and fearful avatars (Trabanelli et al., 2025). When infectious avatars approached participants and reached the so-called peripersonal space, participants were faster at a tactile response task compared with the control conditions. This indicates that the brain anticipates potential contact with pathogens before touch. Moreover, after exposure to infectious avatars, there was a shift in innate lymphoid cells, equivalent to the shift observed after receiving the flu vaccine. Changes in hormone levels related to the hypothalamus-pituitary–adrenal (HPA) axis predicted the activity of innate lymphoid cells, indicating that cognitive appraisal of infection cues seems to recruit the HPA axis to initiate innate defenses (Trabanelli et al., 2025).

There are also strong indications that immune dysregulation and affective symptoms are associated with each other. For example, depressive symptomatology is linked with higher levels of pro-inflammatory cytokines and high C-reactive protein (Frank et al., 2021). Some studies also suggest that elevation in inflammatory markers may precede the onset of depressive symptoms, 2–8 years earlier [reviewed in Drevets et al. (2022)]. In addition, clinical studies have found that anti-inflammatory treatments lower depression symptoms in patients with immunological disorders (Drevets et al., 2022). Aligned with this finding, clinical studies have also found that patients with both major depressive disorder and elevated pro-inflammatory biomarkers may show ameliorations in their depressive symptoms after immune-based treatment, further suggesting the link between immune health and mental health (Drevets et al., 2022).

Inflammation seems to be associated with certain symptoms of depression and not others. A study that included 15 large cohorts of participants with both data on depression and inflammation (blood levels of C-reactive protein and IL6), tested the association between inflammation markers and a large set of affective symptoms. They found that only 5 symptoms of depression showed solid associations with depression, namely, changes in appetite, feelings that everything is an effort, difficulties to keep going or loss of energy, little interest in doing things or feeling unmotivated, and restless sleep (Frank et al., 2021). Of note, these symptoms refer to 4 clusters of symptoms: namely anhedonia, fatigue, or lack of vitality, along with sleep and appetite problems.

Anhedonia is the inability to feel or experience pleasure, leading to a lack of motivation to pursue enjoyable activities, such as seeing friends, or going out. The link between inflammation and anhedonia has been hypothesized to be the result of the effect of pro-inflammatory cytokines on the brain mesolimbic dopaminergic pathways, responsible for reward processing (Drevets et al., 2022). Along with anhedonia, fatigue is another symptom repeatedly associated with immune dysfunction. For instance, people suffering from immune disorders, such as autoimmune diseases (e.g., multiple sclerosis, lupus erythematosus, rheumatoid arthritis), or people suffering from infections, frequently experience strong symptoms of fatigue (Lee and Giuliani, 2019).

In research, inflammation is commonly assessed through circulating markers. These include markers of acute inflammation, such as C-reactive protein or high-sensitivity C-reactive protein, as well as pro-inflammatory cytokines like IL6 and IL1β, or Tumor Necrosis Factor α (TNF-α) (Gabay and Kushner, 1999).

Several lifestyle recommendations have been proposed to optimize immune health. Adherence to anti-inflammatory diet patterns, a nutrition pattern characterized by being rich in fruits, vegetables, whole grains, plant-based proteins, and healthy fats, and by limiting the consumption of red meat and refined sugars, has been associated with lower markers of inflammation (Hébert et al., 2019).

Physical activity is also an important lifestyle intervention with the power to strengthen the immune system. Single bouts of high intensity, high-volume exercise can transiently suppress immune function. Especially in the absence of appropriate recovery, they may give rise to opportunistic infections (Cerqueira et al., 2020). However, regular moderate training seems to enhance immune responses to vaccines, lower the number of senescent T cells, lower the number of proinflammatory cytokines, and increase the performance of natural-killer cells, overall strengthening immune health (Simpson et al., 2015).

### The gut-brain axis

The term “gut-brain axis” refers to the intimate links that exist between the gut ecosystem and the central nervous system, and it is in this context that we cover the interconnections between digestive health and mental health below.

The gut microbiome is the ensemble of living microorganisms (or microbiota) and their products (e.g., metabolites, structural elements, nucleic acids, etc.) that can be found in the human gut (Berg et al., 2020). The human microbiome may be able to influence the nervous system by different mechanisms, such as the production of bacterial metabolites like short-chain fatty acids (SCFAs), tryptophan-derived indoles, or polyamines; the regulation of the production, expression, and turnover of neurotransmitters (such as gamma-aminobutyric acid (GABA), dopamine, and serotonin), and the protection of the intestinal barrier (Carabotti et al., 2015). SCFAs are metabolites that certain types of gut bacteria produce through the fermentation of complex carbohydrates such as dietary fiber (De Vos et al., 2022; Thursby and Juge, 2017). SCFAs play a role in cellular differentiation, proliferation, and apoptosis and they also have an important role in regulating the inflammatory response (Thursby and Juge, 2017). As such, SCFAs could potentially affect the nervous system through the modulation of inflammation (Carabotti et al., 2015). The gut microbiome may also regulate the production of neuromodulators. Certain species of *Bacteroidetes* express the neurotransmitter GABA, which is the main inhibitory neurotransmitter in the brain (Strandwitz et al., 2019). In addition to producing GABA, species of *Bacteroides*, along with the genus *Clostridium* and *Lactobacillus*, from the *Firmicutes phylum*, have a role in the metabolism of tryptophan, by either synthesizing tryptophan or by transforming dietary tryptophan into bioactive compounds, such as the neurotransmitter serotonin (Ruan et al., 2020).

Supporting the possibility that the gut microbiome has a link with mental health, a recent meta-analysis on 23 randomized-controlled trials strongly suggested that probiotic intake can significantly ameliorate symptoms of depression and anxiety (Asad et al., 2024). Stress also seems to change the composition of the microbiota, and it likely does so by eliciting changes in neurotransmitter and pro-inflammatory cytokine levels, which can affect the microbiota directly or indirectly (Konturek et al., 2011).

Dietary patterns are considered to have large effects on the composition of the gut microbiota, although the effects are only detectable in the long term (Wu et al., 2011). For instance, a diet rich in fibers is associated with *Firmicutes* and *Proteobacteria*, while diets that are rich in fat favor the concentration of *Bacteroidetes* and *Actinobacteria* (Wu et al., 2011). A recent study examined how prolonged diet preferences affect the composition and function of the gut microbiota in 5 independent cohorts. Relative to omnivore participants, vegan participants showed an increase of bacteria that are known butyrate producers (a short-chain fatty acid), such as *Lachnospiraceae*, *Butyricicoccus,* and *Roseburia hominis,* which, respectively, are a family, a genus, and a species of *Firmicutes*. Relative to vegans, vegetarians showed higher concentrations of species that are found in cheese and yogurt, such as *Streptococcus thermophilus*, or *Lactobacillus paracasei*, two species of *Firmicutes* which are generally considered beneficial for gut health (Fackelmann et al., 2025). A healthy gut microbiota can have systemic health effects. For instance, specific gut bacteria ferment carbohydrates and convert them to short-chain fatty acids, which play a role in gene expression, cellular differentiation, glucose homeostasis, and the regulation of inflammation (Ruan et al., 2020; Thursby and Juge, 2017).

## Thriving factors: cognitive performance and positive emotionality

### Cognitive performance

Cognitive performance is important for our well-being, and it includes functions such as memory, attention, problem-solving, and decision-making. An optimal cognitive function enables individuals to navigate daily challenges, maintain social relationships, and achieve personal and professional goals, thereby enhancing quality of life.

Conversely, factors like fatigue, sleep deprivation, and stress can affect cognitive performance, leading to symptoms commonly referred to as “brain fog.” This state is characterized by confusion, forgetfulness, and a lack of mental clarity.

Cognitive performance can be affected by the other components of wellbeing and mental health. For instance, fatigue has been associated with diminished cognitive abilities, including reduced attention and slower information processing. Chronic fatigue can lead to persistent cognitive impairments, affecting both personal and professional domains (Ouyang and Luo, 2025). Burnout, a prevalent psychosocial risk factor of demanding working environments, is also associated with reduced general cognitive performance and mental speed (Oosterholt et al., 2014). Sleep deprivation can also disrupt cognitive functions. During sleep, the brain consolidates memories and clears out protein waste products. Lack of adequate sleep impairs memory consolidation, attention, and executive functions (Alhola and Polo-Kantola, 2007).

Cognitive performance can be assessed objectively and subjectively. Objective tests on cognitive skills, and a very non-exhaustive list of the most well-known examples are the Trail Making Test (part B) for cognitive flexibility and divided attention, the Stroop test for inhibitory control, or the Hanoi tower for planning and problem-solving (Lezak, 2004). Self-reported questionnaires can be useful to evaluate the subjective perception of cognitive lapses, and an example of this is the Cognitive Failures Questionnaire (Goodhew and Edwards, 2024).

The following interventions may optimize cognitive performance or help alleviate brain fog.

Engaging in consistent physical exercise has been shown to improve various cognitive functions, including memory, attention, and executive function. A meta-analysis highlighted that long-term aerobic and resistance exercises are particularly beneficial for global cognition and executive function, especially in older adults (Zhang et al., 2023).

Ensuring sufficient and quality sleep is crucial for cognitive function, as it facilitates memory consolidation and protein clearance in the brain. Finally, since chronic stress can impair cognitive functions, stress-reduction techniques such as mindfulness and progressive relaxation may potentially improve mental clarity (van Agteren et al., 2021).

### Positive emotionality

According to the PERMA model of positive psychology (Seligman, 2011), with PERMA standing for Positive Emotions, Engagement, Relationships, Meaning, and Accomplishment, positive emotions are the experience of joy, happiness, love, and gratitude. Positive emotions can increase physical health, they support work and study performance, favor interpersonal relationships, and make us enjoy the present moment. On an important note, and parallel to the recognition of the fundamental role of positive emotions on mental health and wellbeing, we feel it is important to mention here that the promotion of positive emotions should by no means target the elimination or repression of negative emotions such as anger, sadness, or fear. We recognize that negative emotions are evolutionarily adaptive and that experiencing them can lead to growth and creativity.

Lower scores in subjective happiness are associated with stress ratings and with poorer health (Cohen-Louck and Levy, 2023; Liu et al., 2016). While positive emotions have been linked to reduced stress and improved health outcomes, it is equally important to recognize that they are not a panacea for all health issues, and that health problems cannot be simply “wished away.” Overemphasis on maintaining positivity and positive emotions can lead to “toxic positivity,” where negative emotions are dismissed, stigmatized, or ignored, potentially compromising mature emotional processing.

An example of a questionnaire evaluating positive emotions is the Subjective Happiness Scale (Lyubomirsky and Lepper, 1999).

Exercises derived from the practice of positive psychology have shown to be effective in increasing wellbeing and positive emotions, including writing a gratitude journal, where participants are asked to reflect on experiences, events, or people they are grateful for, reflecting about pleasurable experiences, where participants are asked to think about experiences that were fun, amusing, joyful, or that are bringing happiness to their lives (Colombo et al., 2021; van Agteren et al., 2021).

Engaging in mindfulness, which involves present-moment awareness without judgment, has also been linked to improved emotion regulation and increased positive emotions (van Agteren et al., 2021).

## Discussion

The pursuit of mental health and wellbeing is a quest that goes beyond the palliation of misery and psychological suffering. Consequently, it must consider the elements that make life worth living. Here we adopt this view along with the need for mental health to be targeted from a holistic perspective, where, in addition to psychological components, biological aspects of wellbeing and social factors are considered as well. In doing so, we align with the established biopsychosocial framework, while at the same time we emphasize the need to structure these dimensions to guide interventions in mental health and wellbeing.

Interventions aimed at optimizing wellbeing and mental health often lack a theoretical structure. In response to this gap, we have developed a 3-layer framework that puts the focus on 9 components of wellbeing and mental health and delineates their hierarchical relationship between them. It is important to note that this hierarchical structure should be understood as heuristic and hypothesis-generating, rather than empirically established. For instance, our framework can be used to anticipate the range of effects that a given intervention, or a set of interventions, may have on the different components of wellbeing. An important hypothesis that derives from our model is that, due to the close interrelations between the different elements of wellbeing and mental health, interventions aimed at improving one component of wellbeing can yield benefits that extend beyond their initial intended goal, creating a virtuous circle. For example, mindfulness is one of the interventions recommended to ameliorate chronic insomnia and support sleep recovery (Edinger et al., 2021). Mindfulness seems to also be useful for promoting social connections, through facilitating “being present” in social interactions and through an enhancement of positive relations with oneself and the surrounding environment (Zhang et al., 2023). Because mindfulness supports these two foundational elements of wellbeing and mental health, it can be hypothesized that its beneficial effects go beyond them and extend to other layers. Here we cannot disentangle causes from consequences since the data reviewed is observational in nature. However, following the hierarchy of our model, we can hypothesize that by ameliorating sleep and social connections, mindfulness interventions may additionally improve the capacity of an individual to manage daily stressors, and that this in turn may help to increase positive emotionality and cognitive focus. The beneficial effects of mindfulness on all of these domains have already been suggested in the scientific literature (van Agteren et al., 2021).

**Figure 3:**
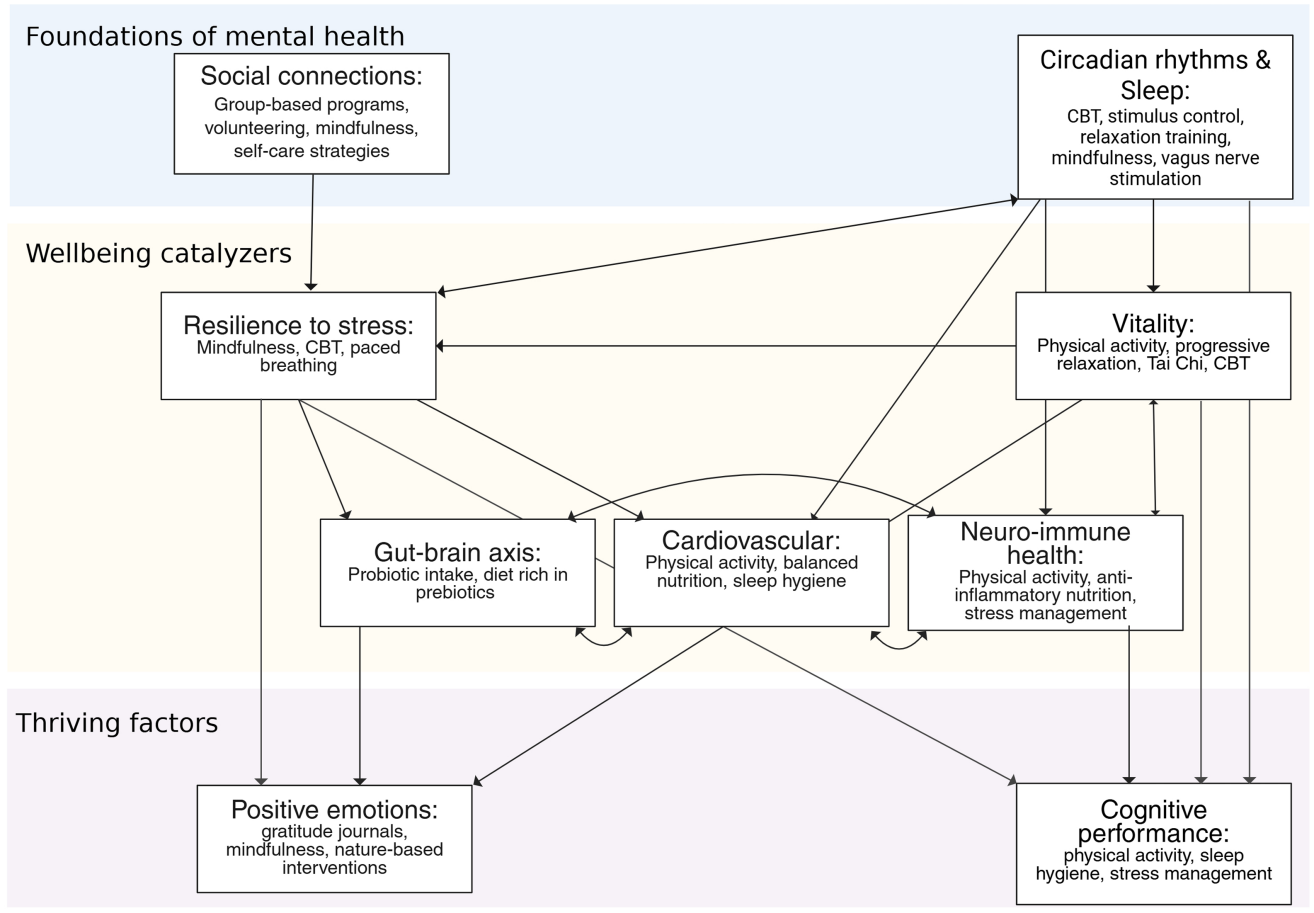
Lifestyle interventions targeting each of the elements of the watershed model of wellbeing and mental health.

The link between physical health and mental health has been well acknowledged in the past, as reflected by the timeless Latin quote “mens sana in corpore sano”. Despite this, biological factors have been left aside in other theoretical frameworks of wellbeing (e.g., Seligman, 2011). There are several health problems, such as digestive issues, cardiovascular disorders, chronic insomnia, or immune dysfunctions, that may be triggered or aggravated by mental health problems such as chronic psychosocial stress, as it has been reviewed above. Here, we also suggest the possibility that interventions to *support* circadian rhythms, immunity, cardiovascular health, and the gut-brain axis may potentially have beneficial effects on certain components of well-being and mental health, notably on positive emotionality and cognitive focus. For instance, physical activity is a long-term protector of cognitive performance by supporting cardiovascular health or neuro-immune health (García-García et al., 2023). In this way, comprehensive interventions should take into consideration physical health dimensions, both as possible indicators of underlying mental health problems and as crucial allies to enhance and protect wellbeing and mental health (Figure 3).

Like other theoretical framework, our model is simplistic, and it leaves aside several other important components of wellbeing and mental health. This is the case for engagement or flow, purpose or meaning, and accomplishment (Seligman, 2011), sexual health, social skills, positive self-perception, or mental health literacy (Fusar-Poli et al., 2020). Future revisions of the framework will consider the inclusion of these and any other aspects of wellbeing that we did not to list here. Similarly, given the vast extent of the available scientific literature, we have not been able to cover the full range of therapeutic options. Some promising interventions may have been missed. As this is a conceptual synthesis rather than a systematic review, the cited literature is necessarily selective. Future work could evaluate the framework using systematic evidence mapping and formal quality assessment. Finally, this paper is purely theoretical and does not provide empirical evidence or intervention studies validating the proposed framework. It is our hope that the watershed framework of mental health and wellbeing proves useful in providing a structure to design comprehensive wellbeing interventions and to track the beneficial effects of these interventions at the different layers of wellbeing and mental health.

## Data Availability

The original contributions presented in the study are included in the article/supplementary material, further inquiries can be directed to the corresponding author.
